# Artificial womb technology and the frontiers of human reproduction: conceptual differences and potential implications

**DOI:** 10.1136/medethics-2018-104910

**Published:** 2018-08-10

**Authors:** Elizabeth Chloe Romanis

**Affiliations:** Centre for Social Ethics and Policy, School of Law, University of Manchester, Manchester, UK

**Keywords:** foetal viability, neonatology, reproductive medicine, embryos and fetuses

## Abstract

In 2017, a Philadelphia research team revealed the closest thing to an artificial womb (AW) the world had ever seen. The ‘biobag’, if as successful as early animal testing suggests, will change the face of neonatal intensive care. At present, premature neonates born earlier than 22 weeks have no hope of survival. For some time, there have been no significant improvements in mortality rates or incidences of long-term complications for preterms at the viability threshold. Artificial womb technology (AWT), that might change these odds, is eagerly anticipated for clinical application. We need to understand whether AWT is an extension of current intensive care or something entirely new. This question is central to determining when and how the biobag should be used on human subjects. This paper examines the science behind AWT and advances two principal claims. First, AWT is conceptually different from conventional intensive care. Identifying why AWT should be understood as distinct demonstrates how it raises different ethico-legal questions. Second, these questions should be formulated without the ‘human being growing in the AW’ being described with inherently value laden terminology. The ‘human being in an AW’ is neither a fetus nor a baby, and the ethical tethers associated with these terms could perpetuate misunderstanding and confusion. Thus, the term ‘gestateling’ should be adopted to refer to this new product of human reproduction: a developing human being gestating ex utero. While this paper does not attempt to solve all the ethical problems associated with AWT, it makes important clarifications that will enable better formulation of relevant ethical questions for future exploration.

## Introduction

In early 2017, news broke of the closest thing to an artificial womb (AW) the world had ever seen. The prototype ‘biobag’ successfully supported lamb fetuses on the current viability threshold. All emerged from the biobag healthy, having seemingly evaded common complications associated with preterm birth.^1^ The biobag facilitates the process of partial ectogenesis: the development of a fetus in an AW during *part of* the gestational period following transfer from the maternal womb.^2^ It shows real promise of a future in which more sophisticated technology could secure better long-term prognoses for premature neonates.

Singer and Wells argued that technologies enabling the artificial gestation of human beings would come about ‘by accident’ as developments in neonatal intensive care (NIC).^3^ Others argue that partial ectogenesis is already a partial reality;^4^ NIC is one of modern medicine’s clearest success stories. Constantly improving technology has supported increasingly premature neonates.^5^ I argue, however, that the biobag is not another improvement in conventional NIC, but an entirely novel approach. The distinctiveness of artificial womb technology (AWT) must be recognised in discussion regarding future clinical applications to prevent harmful decision-making for and by affected parties. Highlighting this distinction is important, as clinicians may easily overlook it because their use of AWT will have the same clinical objective as NIC.

First, I explore the current limitations restricting NIC and prospects for AWT to provide important context for ethical discussion. Second, I argue that AWT will challenge perceptions of viability, a concept referring to the ability of a developing human being to survive ex utero.^6^ AWT, I argue, will most likely be used beyond the current recognised viability threshold (24 weeks from conception) to facilitate partial ectogenesis. During partial ectogenesis, a fetus already developing in utero is transferred to an AW to continue gestating ex utero. This process is distinct from complete ectogenesis (the creation of an embryo using in vitro fertilisation that is gestated entirely in an AW).^2^ The possibility of biobags being used for complete ectogenesis is a more remote possibility and encompasses some distinct ethical issues. Therefore, it is not discussed in this paper. Partial ectogenesis exposes a problem of terminology that will be examined. New terminology should be used to describe the subject of the AW to evade the ethical tethers that existing terms imply, which cloud discussion. The term ‘gestateling’ is introduced to refer to a developing human being in the process of ex utero gestation. Finally, I argue AWT should be treated as conceptually distinct from conventional rescue technologies. I provide three reasons for the distinction: innate differences between the features of AWT and NIC, differences between the subjects of each technology and further potential uses for AWT beyond newborn rescue. While this paper does not attempt to solve all the ethical problems associated with AWT and its experimental use, the crucial clarifications it provides are necessary to consider when formulating relevant ethical questions for future discussion.

## Artificial wombs—where are we?

Preterm birth, before 37 weeks gestation, is the leading cause of death among newborns globally.^7^ The swift advancement of NIC, however, was an example of medicine overcoming some of the biological body’s inherent vulnerabilities. The survival prospects for preterms in developed countries had been steadily improving over the last few decades. So much so that the survival of extremely premature neonates, born at 28 weeks or less,^7^ is no longer wholly irregular. ‘Infant incubators’ have sustained preterms born as early as 21 weeks and 6 days.^8^ However, survival this premature is not the norm. A recent study reported a survival rate of only 0.7% among preterms born at 22–23 weeks.^9^ There is no hope of survival before this point. Preterms on the viability threshold that survive birth often develop complications, resulting in severe disability or death.^8^ In the last 20 years, there has been a 44% increase in preterms born at 22–25 weeks surviving long enough to receive NIC,^10^ but the pattern of mortality and proportion with severe long-term health problems has not meaningfully changed for some time.^11^

### Limitations of neonatal intensive care

The occurrence and severity of complications associated with preterm birth decline markedly with increased gestation.^12^ Neonates born before 26 weeks gestation remain unlikely to survive common complications.^12^ Around 50% of surviving preterms at 26 weeks have a severe long-term impairment. This increases to 75% among those born at 23 weeks.^10^ The biggest issues plaguing preterms include: underdeveloped lungs and respiratory problems, circulatory problems causing low blood pressure and oxygen deprivation and an underdeveloped ability to swallow or suck.^12^ These complications are almost inevitable before 26 weeks. They can be managed by providing mechanical ventilation, administering oxygen, using external pumps to aid circulation and nasogastric feeding.^12^ These functions are all interventions facilitated in infant incubators, and they each carry risks and limitations. Mechanical ventilation and the administration of oxygen can hinder further lung development or damage the lungs.^13^ External aids for circulation can cause heart failure by effecting imbalances in blood flow.^13^ Nasogastric feeding carries a high risk of necrotising enterocolitis (death and leakage of intestinal tissue)^14^ and infection.^12^

Due to the risks and limitations of interventions, some scientists believe the clinical possibilities of NIC have been exhausted.^15^ There is only so much medicine can do for a neonate born without the capacity for an independent life. This is why between 60% and 80% of NIC deaths occur after withdrawal of interventions.^11^ Conventional NIC also raises ethical concerns. When treatment is withdrawn, as is often the case, all treatment achieved was the prolonging of the neonate’s physical suffering and the emotional distress of its parent/s. Possible alternative forms of intervention to those used routinely will still harbour risks and similar barriers to success. With this in mind, researchers are seeking an alternative physiological approach to sustaining underdeveloped human beings by better mimicking the uterine environment to effectively prolong gestation.^i^ 16 This encompasses a support system closer to an AW, facilitating continuing development *as if the neonate had never been born*, as opposed to infant incubators assisting preterms with bodily functions they cannot perform adequately for themselves.

### The biobag

In early testing, the newly designed AW was able to sufficiently mimic the uterine environment to sustain preterm lamb ‘fetuses’ for 4 weeks.^1^ These lambs were developmentally equivalent to human preterms at the recognised viability threshold: 24 weeks. After the incubation period, all subjects were ‘delivered’ and survived. News of the Philadelphia-based team’s success made global headlines.^17^

The biobag consists of a sealed bag to contain the subject, a ‘pump-less oxygenator circuit’ and umbilical cord access. The sealed system prevents outside exposure, minimising the risk of infection. The bag enables constant exchange of amniotic fluid, providing all necessary water and nutrients. Cannulae act as an ‘umbilical cord’ carrying required nutrients and oxygen into the subject’s bloodstream. Circulation is dependent on the subject’s heart working with an oxygenator. This mimics normal placental circulation, ensuring sufficient oxygen and a safe blood pressure.^1^ The biobag effectively simulates natural gestation in utero. All biobag test subjects had continued normal lung development and circulation without any infection.^1^ The three most common complications (lung development, circulation, infection) experienced in NIC appear to have been sidestepped. Another research team based in Australia has also developed an AW system with comparable success in animal studies.^16^ The AW this team has developed, however, is not the focus of this paper because the team are more tentative about the potential clinical application of their technology in humans.^16^ The enormity of these findings, and their capacity to reduce morbidity among preterms, is hard to overstate.

Further refinement of the biobag as well as scientific and safety validation is necessary before clinical use can be anticipated.^1^ It seems probable, however, that we are only several years away from testing on human subjects.^13^ If the results of this animal study are repeated with similar success there will soon be calls for its use from parents trying to overcome troubled pregnancies^18^ or aid preterm children. The biobag study authors identify their ‘clinical target population’ as preterms between 23 and 25 weeks gestation.^1^ The researchers comment that if animal testing continues to yield positive results the morbidity rate among human preterms alone justifies use of the technology.^1^ They are already foreseeing the clinical application of the biobag.^ii^ The team’s brief mention of a justification for experimental use implies they see no meaningful difference between the AW they have designed and NIC relevant to decision-making about its use. It could be argued there is no difference for now, because the objective of the technology’s use will be similar to NIC. However, because of the implications that will be explored the assumption that there is little difference between AWT and current NIC must be critiqued.

## Subjects in the biobag

In this section, I demonstrate how the biobag is likely to be used beyond the current viability threshold, and why this means we need new terminology to describe the subject of an AW. The authors of the biobag study are explicit that their research aims only to reduce incidences of death and disability among ‘just-viable’ preterms. Their objective is not to ‘push back’ the viability threshold,^1^ and they have identified their future clinical target population accordingly. Though the authors do not explain why, narrowing their scope at this stage of experimentation is understandable. Viability is, in many countries, the point at which the fetus is afforded some legal protections limiting abortion access^19^ because, in providing a medicalised model for abortion, viability is a pragmatic compromise between the anti-abortion lobby and pro-choice activists. The researchers may wish to avoid their work becoming embroiled in discussions of broader ethical implications relating to abortion. However, if the biobag is as successful for human preterms as it has been for animals, it will eventually have the effect of changing, at least, perceptions about where the viability threshold lies.

Even with conventional NIC, and its evident limitations, there has been a huge shift in perception regarding when technology should be used to support preterms. Despite the current recognised viability threshold of 24 weeks, and high probability of complications before 26 weeks, rescue is frequently attempted on preterms as young as 22 weeks. Only attempts to resuscitate before 22 weeks are deemed experimental.^20^ There is much societal conditioning encouraging intervention to save preterm babies often regardless of the likely outcome.^5^ The willingness to attempt rescues is the result of clinicians trying their upmost to aid the patient in front of them at the request of the parent/s. When the neonate is only fractionally less developed than preterms routinely sustained, this increases the pressure to attempt rescue. Parents are often willing to challenge clinicians to ensure their premature infant is provided with treatment offering it a chance at life. Challenges sometimes develop into high-profile legal disputes.^21^

Once AWs can ensure the consistent and healthy survival of preterms on the viability threshold, there will be immediate calls, from medical practitioners and parents alike, to use AWT to aid preterms not far behind the current threshold. Similar trends with conventional NIC are how we arrived at the current viability standard. If the biobag works as designed, its subjects will be less likely to suffer complications than if they are supported using conventional NIC. Clinicians will see more value in treatment for younger preterms when AWs are available because outcomes will be better. This willingness to try something different to aid ‘almost surviving’ preterms was ultimately the motivation behind the biobag study. It is unlikely that placing younger subjects in AWs would be seen as controversial, with little opposition to attempts, if AWT is successful for older neonates.

The potential use of AWs, which challenges our understanding of viability, exposes a terminology problem. The human being growing in the AW is in the process of artificially induced gestation. It will, in some cases, be incapable of exercising any independent capacity for life and be more ontologically similar to the pre-viability fetus in utero, than to what is thought of as a ‘newborn baby’. The terminology used to describe preterms is similar to that for newborns at full-term. Calling the human being gestating ex utero a ‘preterm’ or ‘newborn’ is arguably misleading as to its behaviour and the extent of its development. This will be explored further when comparing AWT and NIC. Notably, the biobag team refer to their subjects as fetuses. Describing the human being gestating ex utero in the AW as a fetus, in an attempt to distinguish it from a neonate receiving NIC, is also confusing and misleading. Most medical definitions of the fetus imply it is located inside a human gestator by describing it as ‘unborn’.^22^

The terms used to describe preterms and fetuses are inappropriate in this context and so a different term, which avoids the connotations of using either ‘newborn’ or ‘fetus’, is needed. I will, therefore, refer to the human being in the AW as the ‘gestateling’. This term provides useful clarity and an accurate descriptor for the AW subject. A gestateling is a human being in the process of ex utero gestation exercising, whether or not it is capable of doing so, no independent capacity for life. The gestateling might soon, through experimental treatment, become a medical reality complicating ethico-legal discussion in obstetrics and neonatology.

## Beyond just another form of intensive care

In this section, I advance three justifications for treating AWT as distinct from NIC. There is noticeably a paucity of academic commentary on this matter. However, this investigation is necessary to determine how biobag trials with human subjects can begin in an ethical manner. Singer and Wells argued AWT would be just an extension of conventional NIC. Therefore, experimentation would be inherently ethical. By only extending existing interventions, it would not be reckless with human life, but consist of connected medical treatments undertaken to aid a particular patient.^3^ However, AWT is potentially emerging as the only feasible technology to erode the current viability threshold, despite intentions to the contrary. Many scientists believe conventional NIC has ‘hit a wall’ that will seemingly always hinder its ability to support younger preterms.^15^ AWs, having different innate features, are more radical in approach. The three reasons for treating AWT as distinct I defend are relevant in considering future clinical applications of AWT and how we might treat affected parties. Harmful decision-making by or on behalf of parties involved is likely if conceptual differences are ignored.

### The innate features of AWT

When two medical technologies provide the same function they can be treated as interchangeable, unless the process of each markedly distinguishes them. Medical and surgical remedies, for example, are distinguished by comparative invasiveness. AWT and conventional NIC both support underdeveloped humans. Hendricks observes, however, that AWT is different in nature because it provides more comprehensive support.^23^ Current care is dependent on the preterm ‘tolerating artificial ventilation’, which is limited by a natural threshold of lung development. This threshold does not limit the AW because it better resembles natural gestation,^23^ and thus does not rely on the lungs for gas exchange. There appears to be no natural limit, at least related to lung development, restricting the AW.

The inherent difference between AWT and NIC is more nuanced than its effect on one aspect of development. AWT has the capacity to entirely replace a human function: it works by replicating and replacing a biological process, rather than attempting a rescue. This makes it, in effect, a move into the realm of automation. The purpose of AWT is to treat a gestateling *as if it had never been born,* and thus requires the gestateling to exercise, regardless of its capabilities, no independent capacity for life. The traditional infant incubator, in contrast, has the purpose of only supporting what capacity for life the newborn is already exercising or beginning to exercise. Therefore, the neonate shoulders some of the burden of sustaining itself. The gestateling, however, has no such pressure incumbent on them during partial ectogenesis. AWT requires of its subject *no exercise of any independent capacity for life*. If the AWT were switched off or malfunctioned, an underdeveloped gestateling would die just as a fetus would in utero during a severe placental abruption. The underdeveloped premature neonate in an infant incubator that is then switched off, however, might survive a short time before life functions dwindled. AWT is closer to technologies sustaining individuals with brain stem death, than to forms of artificial support provided to comatose patients with working nervous systems still coordinating some important bodily functions. The latter is more comparable to NIC.

Rieder argues that when physicians, at present and with only current technologies available, engage in the resuscitation and treatment of extremely premature infants they effectively take over the process of creating them.^24^ He argues that conventional NIC does not rescue when used to aid extremely premature infants, but attempts to uptake the creative process and ‘artificially continue gestation’. He posits this observation needs little defence.^24^ It is my contention, however, that his observations work when applied to technologies like the biobag, but are misplaced when applied to conventional NIC. Rieder conflates a human being continuing to develop with gestation (the creative process). Gestation, whether in or ex utero, is distinct from ‘continuing to develop after being born’. Human beings continue to develop long after the gestational process is complete, for example as part of development continuing into childhood.^25^ Gestation, however, is different in that it is a process of formation, which if not completed adequately the human being has no capacity for life independent. The limitations of conventional NIC demonstrate it is not capable of anything other than providing assistance with life functions a preterm is struggling to perform itself. Conventional NIC is not a ‘creative process’, AWT, however, is.

A further distinction between AWT and conventional NIC is in environment. Intensive assistance with life functions is wholly invasive, and yet also leaves the preterm exposed and within an environment where some human interaction (skin-to-skin contact) is possible. The gestateling in the biobag, however, is encased and support is almost entirely non-invasive. Support mechanisms surround rather than aggressively invade the gestateling. One imagines the process of AWT would be less stressful and painful for the developing human. Less is required of, and there is less to disturb, the gestateling. Notably, AWT is so unique in method that outcomes will be different and, outside of one study, are unknown. There is no way of knowing now, or anytime soon, what the long-term implications of artificial gestation might be. Gestatelings may be subjects of a research trial long after removal from the biobag. For some time, the biobag will remain a new experimental treatment, while conventional NIC is business as usual. The unknown implications should encourage some caution.

### The subject of partial ectogenesis

AWT could in theory, and possibly in practice, support a gestateling unable to exercise any independent life functions ex utero *at all*. The marked difference in terms of gestational age, and consequent abilities, of the subject potentially sustained by each support mechanism is striking. Hendricks argues the status of the subject would, therefore, be an important distinguishing factor. We may be inclined to accept that AWT is different because it can sustain something that ‘does not look like a baby’.^23^ Whether there is any difference in moral status between the gestateling and the premature neonate is beyond the scope of this paper. There is, however, based on their dissimilarities in development and appearance, a huge difference in their capacities. This difference is meaningful in terms of the function technology must serve to sustain them.

Let us assume AWT does not confuse the viability threshold further and it has the same natural limitations as NIC in terms of the subjects it could support.^iii^ There remains a distinction in the way the subjects behave. The significance of the subjects’ contributing, or not, to their own survival has already been highlighted. Additionally, the premature neonate is available for social interaction, can experience the benefits of connection with other human beings and become embedded in social networks. Other individuals can interact directly and physically with it. The gestateling is shut off from the outside world and does not touch, smell or interact with anything other than its artificial gestator. This isolation will influence the perception of and, on occasion, the feeling attached to each entity. These perceptions will impact, in various meaningful ways, on the decision-making of those surrounding the gestateling.

### Potential uses of partial ectogenesis

Finally, AWT introduces opportunities beyond more efficacious care for preterms. Partial ectogenesis, once AWs are available, could become a distinct course of action in obstetrics to manage dangerous pregnancies. A dangerous, but wanted, pregnancy is not wholly uncommon and the choices at present seem painfully bleak. When pregnancy threatens a woman’s life, she is usually advised to have an abortion. The alternative is that she continues the pregnancy hoping she survives long enough to deliver a healthy child, but taking the risk that neither she, nor her fetus, will survive. In 2016, Heidi Loughlin faced this decision after being diagnosed with a cancer untreatable during pregnancy. Loughlin refused to accept there was no alternative to choosing between her life and her fetus. She elected for a third choice: remaining pregnant until 28 weeks and opting for premature delivery. Unfortunately, the premature neonate did not survive long after delivery.^26^ This decision was partial ectogenesis in action, but before AWT was available. An AW, were it accessible, may have changed the odds. AWT might encourage more women with dangerous pregnancies to make Loughlin’s choice. It might even be that, in future, women wish to opt for AWT over continuing pregnancy in situations of less concern, for example to avoid unpleasant symptoms like morning sickness. AWT is, therefore, distinct from rescue technologies because it introduces the possibility of the extraction of gestatelings that would never have existed ex utero otherwise. AWT in these situations performs the function of reliably sustaining gestatelings removed from pregnant women and unable to sustain themselves. However, AWT is then functionally not just about ‘sustaining a fetus/gestateling’ but enabling pregnant women to choose an alternative to pregnancy without risking the loss of the product of reproduction. NIC, because of its risks and limitations, will never be considered a reliable enough alternative to gestation to enable this choice.

If AWT is chosen as an alternative to the ‘dangerous pregnancy-abortion’ dichotomy, an extraction procedure will be part of the therapeutic process and is also experimental. A safe method of extraction, potentially a ‘more complex and intricate’ C-Section must be developed^27 28^ inevitably involving trial and error. We are considering experimenting on gestatelings and also potentially considering experimenting on the women who carried them.

## Conclusion

The possibility of gestation ex utero begins with technology initially used as an alternative to conventional NIC before inevitably challenging the viability threshold. The biobag, however, is more than a mere extension of conventional preterm care. It marks a shift in physiological approach for three reasons.

First, AWT replaces a natural function rather than facilitating a newborn rescue. Instead of assisting a premature neonate with functions it is struggling to perform alone, AWT treats its subject *as if had not been born*. Unlike a preterm in intensive care does, the gestateling does not have to exercise any independent capacity for life. AWT also places the gestateling in a different environment, the consequences of which are still unknown. Second, if testing on human ‘just-viable’ preterms is successful, the technology is likely to be used beyond the current viability threshold. Clinicians and parents have incentives to try AWT to sustain preterms only slightly below the threshold, shifting perceptions of viability. Thus, AWT could sustain subjects with very different capacities. These subjects cannot be appropriately described as either babies or fetuses because they are unique in behaviour, location and the process they are undergoing. The term ‘gestateling’ should be used to identify the developing human being in the AW. Third, AWs have potential clinical uses beyond conventional rescue technologies. AWT might appear just a better alternative to NIC, but its development is more significant and will enable the birth of partial ectogenesis as a therapeutic process in itself.

Identifying these distinctions is crucial to inform ethicolegal discussion and ensure better protection for affected parties. Recognising the difference in subject, and terminology that might helpfully be deployed to describe it, will also prevent the interference of value laden terms and bring clarity to this discussion. Being mindful of these differences allows us to consider what, if any, additional regulation is appropriate to ensure AWT research and its potential clinical applications are ethical.

## Footnotes

i A research team based in Australia attempting to design an AW system similar to the biobag explicitly acknowledges this shift in their approach.

ii It should be noted that while the technology has shown significant promise, the experiments were conducted on living beings with a different physiology to human beings. The success of the project does not necessarily mean that the biobag will be as successful when used to attempt to save human preterm neonates. The researchers, however, do imply there is enough reason to believe the technology might successfully be used on human subjects to justify experimental application on human beings in future.

iii It is worth emphasising, given the reasoning advanced elsewhere in this paper that this is a big and unwarranted assumption to make.

## References

[1] Partridge E, Davey M, Hornick M, et al An extra-uterine system to physiologically support the extreme premature lamb. Nat Commun 2017;8:1–15. 28440792 10.1038/ncomms15112 PMC5414058

[2] Gelfand S, Shook J Ectogenesis; artificial womb technology and the future of human reproduction. New York, Rodopi, 2006.

[3] Singer P, Wells D Ectogenesis : Shook J, Ectogenesis; artificial womb technology and the future of human reproduction. New York, Rodopi, 2006:9–25.

[4] Cannold L Women, ectogenesis and ethical theory : Gelfand S, Shook J, Ectogenesis; artificial womb technology and the future of human reproduction. New York, Rodopi, 2006:47–58.

[5] Simonstein F Artificial reproduction technologies (RTs) - all the way to the artificial womb? Med Health Care Philos 2006;9:359–65. 10.1007/s11019-006-0005-4 16988897

[6] Glover J Causing death and saving lives. London: Penguin Books, 1990.

[7] Azad K, Mathews J Preventing newborn deaths due to prematurity. Best Pract Res Clin Obstet Gynaecol 2016;36:131–44. 10.1016/j.bpobgyn.2016.06.001 27545716

[8] Alghrani A Regulating the reproductive revolution: ectogenesis – a regulatory minefield? : Freeman M, Law and bioethics: volume 11. Oxford: Oxford University Press, 2008:303–32.

[9] Pierrat V, Marchand-Martin L, Arnaud C, et al Neurodevelopmental outcome at 2 years for preterm children born at 22 to 34 weeks' gestation in France in 2011: EPIPAGE-2 cohort study. BMJ 2017;358:j3448 10.1136/bmj.j3448 28814566 PMC5558213

[10] Moore T, Hennessy EM, Myles J, et al Neurological and developmental outcome in extremely preterm children born in England in 1995 and 2006: the EPICure studies. BMJ 2012;345:e7961 10.1136/bmj.e7961 23212880 PMC3514471

[11] Costeloe KL, Hennessy EM, Haider S, et al Short term outcomes after extreme preterm birth in England: comparison of two birth cohorts in 1995 and 2006 (the EPICure studies). BMJ 2012;345:e7976 10.1136/bmj.e7976 23212881 PMC3514472

[12] Lissauer T, Clayden G Illustrated textbook of pediatrics. London: Mosby Elsevier, 2012.

[13] Couzin-Frankel J Fluid-filled ‘biobag’ allows premature lambs to develop outside the womb. Science 2017;25 10.1126/science.aal1101

[14] Great Ormond Street Hospital for Children NHS Foundation Trust. Necrotising enterocolitis. 2016 http://www.gosh.nhs.uk/medical-information/search-medical-conditions/necrotising-enterocolitis (accessed 7 Nov 2017).

[15] Hendricks J Not of woman born? Technology, relationship and right to a human mother. *College of Law Faculty Scholarship* . 2011 http://trace.tennessee.edu/utk_lawpubl/45 (accessed 28 Feb 2018).

[16] Usuda H, Watanabe S, Miura Y, et al Successful maintenance of key physiological parameters in preterm lambs treated with ex vivo uterine environment therapy for a period of 1 week. Am J Obstet Gynecol 2017;217:457.e1–457.e13. 10.1016/j.ajog.2017.05.046 28646647

[17] Roberts M Premature lambs kept alive in ‘plastic bag’ womb, BBC News. 2017 http://www.bbc.co.uk/news/health-39693851

[18] Ectogenesis WJ Liberation, technological tyranny or just more of the same? : Gelfand S, Shook J, Ectogenesis; artificial womb technology and the future of human reproduction. New York, Rodopi, 2006:129–38.

[19] Roe v Wade 410 U.S. 113. 1973. 12038376

[20] Nuffield Council on Bioethics. Critical care decisions in fetal and neonatal medicine: ethical issues: Latimer Trend & Company, 2006.

[21] Great ormond street hospital nhs foundation trust v yates and others (No 2) EWHC 1909 (Fam). 2017.

[22] Martin E Concise medical dictionary. Oxford: Oxford University Press, 2015.

[23] Hendricks J Not of woman born: a scientific fantasy. Case Western Reserve Law Rev 2012;62:399–445.

[24] Rieder TN Saving or creating: which are we doing when we resuscitate extremely preterm infants? The American Journal of Bioethics 2017;17:4–12. 10.1080/15265161.2017.1340988 28768134

[25] Hayden D, Wilkinson D Asymmetrical reasons, newborn infants, and resource allocation. The American Journal of Bioethics 2017;17:13–15. 10.1080/15265161.2017.1341000 28768136

[26] Walker P Baby delivered early to allow mother’s cancer treatment dies. The Guardian 2015;20.

[27] Murphy J Is pregnancy necessary? Feminist concerns about ectogenesis : Gelfand S, Shook J, Ectogenesis; artificial womb technology and the future of human reproduction: New York, Rodopi, 2006:27–46.

[28] Schultz J Development of ectogenesis: how will artificial wombs affect the legal status of a fetus or embryo. Chic-Kent Law Rev 2010;84:877–906.

